# High Incidence of Partial Biotinidase Deficiency in the First 3 Years of a Regional Newborn Screening Program in Italy

**DOI:** 10.3390/ijerph19138141

**Published:** 2022-07-02

**Authors:** Daniela Semeraro, Sara Verrocchio, Giulia Di Dalmazi, Claudia Rossi, Damiana Pieragostino, Ilaria Cicalini, Rossella Ferrante, Silvia Di Michele, Liborio Stuppia, Cristiano Rizzo, Francesca Romana Lepri, Antonio Novelli, Carlo Dionisi-Vici, Vincenzo De Laurenzi, Ines Bucci

**Affiliations:** 1 Center for Advanced Studies and Technology (CAST), “G. d’Annunzio” University of Chieti-Pescara, 66100 Chieti, Italy; daniela.semeraro@unich.it (D.S.); sara.verrocchio@unich.it (S.V.); giuliadd@gmail.com (G.D.D.); claudia.rossi@unich.it (C.R.); damiana.pieragostino@unich.it (D.P.); ilaria.cicalini@unich.it (I.C.); rossella.ferrante@unich.it (R.F.); liborio.stuppia@unich.it (L.S.); vincenzo.delaurenzi@unich.it (V.D.L.); 2 Department of Medicine and Aging Science, “G. d’Annunzio” University of Chieti-Pescara, 66100 Chieti, Italy; 3 Department of Psychological, Health and Territory Sciences, School of Medicine and Health Sciences, “G. d’Annunzio” University of Chieti-Pescara, 66100 Chieti, Italy; 4 Department of Innovative Technologies in Medicine and Dentistry, “G. d’Annunzio” University of Chieti-Pescara, 66100 Chieti, Italy; 5 Department of Pediatrics, “Spirito Santo” Hospital, 65124 Pescara, Italy; silvia.dimichele@asl.pe.it; 6 Division of Metabolism, Bambino Gesù Children’s Research Hospital, 00165 Rome, Italy; cristiano.rizzo@opbg.net (C.R.); carlo.dionisivici@opbg.net (C.D.-V.); 7 Translational Cytogenomics Research Unit, Bambino Gesù Children’s Hospital, IRCCS, 00165 Rome, Italy; francescaromana.lepri@opbg.net (F.R.L.); antonio.novelli@opbg.net (A.N.)

**Keywords:** newborn blood spot screening, biotinidase deficiency, expanded newborn screening, inborn errors of metabolism, biotinidase gene variants, biotinidase activity

## Abstract

Biotinidase deficiency (BD) is an autosomal recessive inherited disorder in which the enzyme biotinidase is totally or partially defective and the vitamin biotin is not recycled. BD meets the major criteria for a population screening program. Newborn bloodspot screening (NBS) allows early diagnosis of BD, thus preventing the high morbidity and mortality associated with untreated disease. Both profound and partial BD variant can be detected by NBS test, and serum enzyme activity and/or mutational analysis are required for definitive diagnosis. In Italy, BD is included in the screening panel for inborn errors of metabolism (IEMs) that has been declared mandatory in 2016. We analyzed the data of the first 3 years of the NBS for BD in our region (Abruzzo, Italy), with the aim to describe the outcomes of this recently introduced screening program. In over 26,393 newborns screened, we found 2 carriers and 16 cases with genotype associated with partial BD. Since the serum biotinidase assay has been recently introduced in our algorithm, only three of our newborns met the criteria of genetic and biochemical confirmation, with an incidence of 1:8797, which is in the high range of what has been reported in the literature. All affected infants carried the 1330G>C (D444H) variant in compound heterozygosis, with variants known to be associated with profound BD. A variant previously not described and likely pathogenic was found in one newborn. None of the infants had signs or symptoms. The study of the distribution of the enzyme activity in our population allowed us to validate the adopted cutoff with which the program has a positive predictive value of 18% and to analyze some preanalytical factors influencing biotinidase activity: A correlation of the enzyme activity with gestational age and time at specimen collection was found. Lower mean values of enzyme activity were found in infants born in the summer.

## 1. Introduction

The cytosolic enzyme biotinidase (BTD) liberates free biotin from biocytin during the proteolytic turnover of holocarboxylases and other biotinylated proteins [1] Biotin, an essential water-soluble B vitamin, is the coenzyme of four carboxylases that are involved in fatty acid synthesis, amino acid catabolism, and gluconeogenesis [2]. Biotinidase deficiency (BD) is an autosomal recessive inherited disorder caused by BTD gene variants, resulting in absent or reduced activity of the enzyme [3]. BTD gene has been mapped to the chromosome 3p25, and more than 200 variants of the gene have been reported [4]. Most of them cause a loss of more than 90% of BTD activity, while the most frequent variant (p.D444H) results in about 50% loss of enzyme activity [5]. According to the entity of the residual enzyme activity, a dichotomous functional definition is used to distinguish profound and partial BD (BTD activity below 10% and equal to 10–30% of mean of serum activity of normal individuals, respectively) [6,7]. The incidence of BD varies across countries. Earlier studies indicated an overall incidence of 1 in 60,000 newborns worldwide [8], but the increasing inclusion of BD in the national screening programs will probably change the incidence rate figures [6,8]. Changes in the method for BTD activity assay from colorimetric to the more sensitive fluorometric can also lead to increased detection rate of the screening programs over time [9]. In the more recent studies, a higher incidence has been reported in some countries, such as Brazil, Turkey, and Italy [6,10,11,12,13,14]. The clinical presentation of BD is variable mostly in relation to the degree of the residual enzyme activity, although different expressions in a family with the same genotype has been reported [5,6]. Classically, patients with profound BD exhibit seizures, hypotonia, skin rash, or alopecia. Ataxia, developmental delay, visual problems, and hearing loss are reported in more than 50% of affected individuals [3]. If affected patients are not treated with biotin, metabolic decompensation, coma, or death can occur. Symptoms usually appear between 2 and 5 months of age but may not be evident until later in childhood or during adolescence; several cases of asymptomatic adults with profound BD have been also reported [15]. The occurrence of clinical manifestations in subjects with partial BD is still debated, since it is limited to a few individuals presenting with mild cutaneous symptoms, non-specific neurological signs (hypotonia, seizures, autism, developmental delay, etc.), or hearing problems [6,11]. In asymptomatic patients, neurocutaneous symptoms might appear during stress such as infections or starvation. In profound BD, some symptoms, e.g., neurosensorial deafness and optic atrophy, may be irreversible even under biotin treatment [16]. Biotin supplementation at pharmacological doses (5–20 mg daily) prevents the development of symptoms in children with profound BD and improves neurological and cutaneous features in symptomatic patients [17]. Treatment of partial BTD is controversial; reports of untreated patients who developed symptoms and the lack of toxicity of the vitamin favor treatment of partial BTD patients with 1–5 mg of oral biotin per day [6,11,18]. The high morbidity and mortality associated with untreated profound BD and the effectiveness of early biotin treatment in preventing most of the symptoms make BD suitable for inclusion in newborn bloodspot screening (NBS) programs. Moreover, the population incidence is comparable to that of other disorders screened, and an inexpensive and reliable screening test is available [5]. Following the development of a colorimetric method to determine BTD activity in dried blood spots (DBS), BD has been incorporated into the screening program in the United States and in many countries around the world [5,16]. Infants with a positive screening test undergo clinical evaluation and measurement of BTD activity in serum and/or genetic analysis. In Italy, BD is one of the disorders of the primary core panel of NBS for inborn errors of metabolism (IEMs) that includes also testing for amino acid and urea cycle, fatty acid oxidation, organic acid disorders, and galactosemia. The program has been declared mandatory in 2016 by specific legislations and has been added to the ongoing screening program for phenylketonuria, congenital hypothyroidism, and cystic fibrosis. We reviewed the data of the first 3 years of NBS for BD performed at our laboratory for the Region of Abruzzo in Italy with the aim to evaluate the incidence of BD; to describe the biochemical, clinical, and mutational findings; and to analyze the performance indicators of this newly introduced screening algorithm.

## 2. Materials and Methods

### 2.1. Study Population and Screening Protocol

We retrospectively analyzed data of infants who underwent NBS for BD, as a part of the nationwide expanded newborn screening for IEMs, in the region of Abruzzo, Italy, between the setting out of the program, from December 2018 to December 2021. The screening was performed at the Center for Advanced Studies and Technology (CAST) of the University of Chieti-Pescara, the regional reference laboratory. According to the Italian NBS core panel, the laboratory screens all newborns (birth rate around 9.000/year) for 38 IEMs, as well as for congenital hypothyroidism (CH) and cystic fibrosis (CF). Blood samples were obtained from newborns 48–72 h after birth by a heel prick, and dried blood spots (DBS) were collected on a filter paper (Ahlstrom 266). Collection of two or three specimens was scheduled for preterm, low-birth-weight, transfused, on parenteral nutrition or sick newborns as well as for newborns transferred to another hospital before 48 h of life. Screening test for BD was performed by measuring BTD activity in DBS samples by a semi-quantitative time-resolved immunofluorescence method on a fully automated integrated screening plate processor (GSP^®^ Neonatal Biotinidase kit, PerkinElmer, Wallac Oy, Turku, Finland). At the set-up of the program, the preliminary cutoff of the enzyme activity was set at 85 U/dL (0.5 percentile of the results of the kit manufacturer’s studies). For any result below 85 U/dL, a retest was performed in duplicate in the same DBS and, if confirmed abnormal, a recall sample was requested. If the BTD activity at the repeat test was still below the cutoff, the case was reported as “positive,” i.e., suspected of BD deficiency, and notified to the medical team. In addition to the periodical cutoff recalculation to evaluate any significant deviation of the 85 U/dL from the lower percentiles of our population, the analysis of the distribution of biotinidase activity in a large sample of newborns (described in Section 2.3) as well as data coming from the diagnostic confirmation was used to validate the cutoff. All DBS of the newborns were also analyzed by tandem mass spectrometry (MS/MS) for the quantification of amino acids and acylcarnitines as well as by fluorescence-based assay for thyroid stimulating hormone (TSH), immunoreactive trypsin (IRT), and total galactose (TGal), as required by the national NBS protocol.

### 2.2. Diagnostic Confirmation

Positive-screen-test newborns were referred to the Pediatric Department of the Santo Spirito Hospital in Pescara. Clinical evaluation included general and neurological examination. The follow-up included assessment of psychomotor development, auditory and visual functions, and dermatological problems. On the day of first clinical consultation, a DBS specimen for BTD activity assay was collected from both the parents and siblings in the 16 families of the positive newborns.

After genetic counseling, molecular analysis was carried out on screening-positive newborns and on their parents. Coding regions as well as the exon/intron junctions of the *BTD* gene were sequenced with Twist Custom Panel (Clinical Exome—Twist Bioscience) on platform NovaSeq 6000 (Illumina, San Diego, CA, USA).

Measurement of BTD activity in serum has been introduced recently in the screening algorithm. A blood sample was collected from four infants at the clinical reference center and sent in dry ice to the NBS laboratory. Serum BTD activity was measured by a colorimetric assay using the N(+)-biotinyl-4-aminobenzoic acid (B-PABA) as the substrate (Biotinidase serum/plasma, LTA Milan, Italy): normal value 4.4–12 nmol/min/mL; partial BD 0.7–2.1 nmol/min/mL; and heterozygous 2.2–5.2 nmol/min/mL. A positive control and a serum sample from a normal individual were contextually analyzed.

### 2.3. Analysis of BTD Activity Value Distribution

De-identified data of all the screening test results were extracted from the laboratory computerized records, entered in a FileMaker database (FileMaker Pro Advanced 14.0.1, Inc., Santa Clara, CA, USA), and then analyzed using the statistical software Stata (Stata 15.1, College Station, TX, USA). Distribution of BTD activity at the initial screening test in 11,788 full-term, normal-weight newborns who were not admitted to neonatal intensive care units was analyzed. Normality of BTD activity values was assessed through the Shapiro–Wilk test. Mean, standard deviation, median, interquartile range, and lower percentiles (1%, 0.5%) were calculated. The values were compared with the 18 positive samples referred for diagnostic confirmation. In the whole newborn population, relationships between DBS biotinidase activity, gestational age, birth weight, and age at blood collection were evaluated using Spearman’s rank-order correlation coefficient. Seasonal variations in BTD activity in samples collected at 48–72 h were analyzed with the Kruskal–Wallis test. The two-sample Wilcoxon rank-sum test was used to evaluate the differences between the values observed in the summer and in the other seasons as well as between full-term normal-birth-weight (≥37 weeks gestational age ≥2500 g) and preterm low-birth-weight newborns.

## 3. Results

### 3.1. Screening Outcome

From December 2018 to December 2021, in all, 26,393 newborns were screened. At the initial screening test, none of the infants had BTD activity close to the limit of detection of the assay (14 U/dL), suggestive of profound BD. Below cutoff, BTD activity was found in 88 newborns, who were recalled for a repeat specimen (recall rate 0.33%). Among them, there were 6 preterm/low-birth-weight newborns. At the repeat screening, BTD activity was fully normal in 63 of the recalled newborns. A third specimen was required for 7 newborns with borderline results, and normal values of activity were reported at the repeat screening test for all of them. In 18 newborns, low BTD activity was confirmed at the repeat screening test and all were referred for clinical evaluation and diagnostic confirmation. 

The results of amino acid and acylcarnitine analysis on tandem mass (MS/MS) were unremarkable, as well as those of TSH, IRT, and TGal assays.

### 3.2. Diagnostic Confirmation and Clinical Management

At the time of the first clinical consultation, no symptoms as well as signs related to the disease were reported in all the cases. None of the 18 families were of consanguineous marriage. Family screening revealed in two distinct families one parent and one sibling, both asymptomatic, as having partial BD. At recall for confirmatory diagnostic testing, the profiles of amino acid and acylcarnitine analysis on tandem mass (MS/MS) and of urinary organic acid GC/MS analysis were unremarkable in all subjects.

Molecular analysis of the BTD gene revealed variants associated with partial BD in 16 infants, among them 2 siblings born 20 months apart. The remaining 2 were carriers harboring a single variant. All the 16 infants carried the 1330G>C (D444H) variant in compound heterozygosis with c.1368A>C (p.Q456H) in 5, with c.755A>G (p.D252G) in 3, and with other previously described as associated to partial BD in 8; a new variant (p.F250L) not described, and likely pathogenic by prediction tools, was found in 1 infant; Table 1.

Preliminary data on serum BTD activity assay are available: in 2 infants with genetically confirmed partial BD, the serum assay substantially confirmed the DBS screening test. One of these infants carried the second-most-common compound heterozygous alleles of our population (c.1330G>C/c.755A>G). In a third infant, serum biotinidase activity in the range of partial BD was confirmed in a laboratory outside our region. In addition, the two carriers had values in the range of heterozygotes according to the reference value of the method.

Based on the DBS screening test and on the genetic analysis, 16 newborns were diagnosed with partial BD, with an incidence of 1:1649. Nevertheless, serum enzyme assay was available only for three infants. Therefore, based on genetic and biochemical confirmation, the incidence of partial BD was 1:8797 newborns screened.

All the infants underwent biochemical and clinical workup: blood count with formula, biochemical profile with lactate, ammonia, blood gas analysis, audiometry, eye examination, growth, and neurological development evaluation as well as skin examination. None of them presented signs or symptoms related to BD at first clinical evaluation as well as at the follow-up. Psychomotor development and auditory and visual functions were normal, and no relevant dermatological problems attributable to BD were detected.

At the beginning of the program, we recommended biotin therapy with 5 mg/day when the children were stressed, such as during infection or after vaccination.

No child had complications clearly related to partial BD at follow-up. One patient developed a mild metabolic acidosis during acute Rotavirus gastroenteritis, quickly corrected with intravenous rehydration. Another patient presented mild language delay at 2 years, but it is difficult to assert if it was caused by BD. In one newborn, in a repeat follow-up screening test at the age of 16 months, low biotinidase activity was confirmed, while on MS/MS analysis, a higher-than-cutoff value of methylmalonyl-3-hyroxy-isovalerylcarnitine C4DC-C5OH and a significant alteration in its ratio with short and medium chain acylcarnitines were observed. A similar profile was observed in the DBS MS/MS analysis of the proband’s father [18].

After a careful clinical and biochemical evaluation based on careful review of the data reported in the literature, starting from 2021, we decided to treat with biotin 5 mg daily all infants with confirmed partial BD.

Metabolic clinical management, genetic counseling, and follow-up of all diagnosed patients were provided by our team of clinicians and geneticists. All infants visited the clinical reference center every 12 months for clinical and biochemical follow-up. To date, all BTD patients are asymptomatic.

### 3.3. BTD Activity Values Distribution

**Figure 1:**
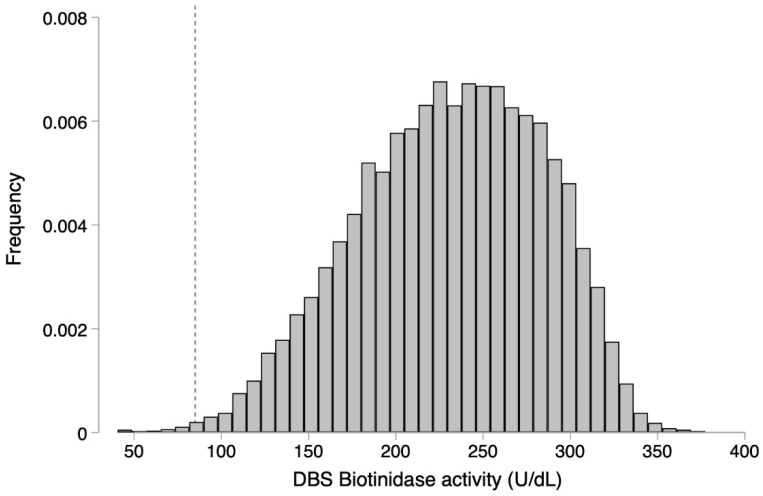
DBS biotinidase activity distribution in all the newborns screened. The dotted line represents the 85 U/dL cutoff.

The biotinidase activity distribution of all the newborns screened is illustrated in Figure 1.

**Figure 2:**
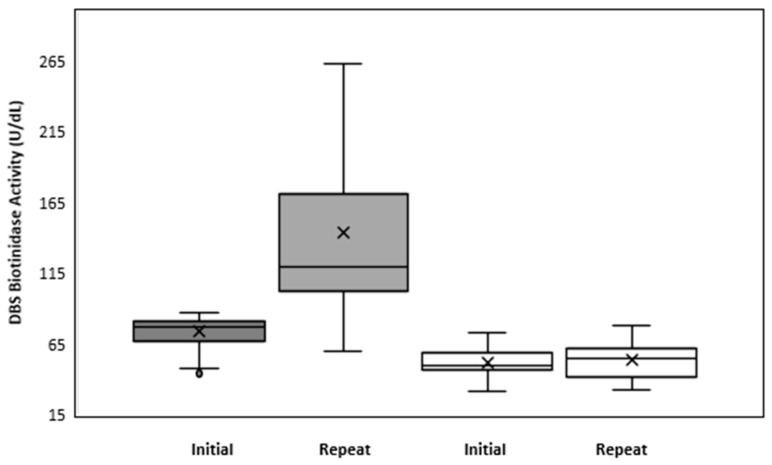
DBS biotinidase activity in false positives (gray boxes) and true positives (white boxes) at the initial and repeat DBS screening test.

The BTD activity values in a representative sample of 11,788 at term, normal-weight newborns with specimens collected at 48–72 h of life followed a non-normal distribution. The percentile calculation showed: 50% 236.15 U/dL, 1% 108.1 U/dL, and 0.5% 96.37 U/dL. The mean and the standard deviation of DBS biotinidase activity was 232.3 ± 52.8 U/dL; the median and the interquartile range were 236.15, and 78.9 U/dL, respectively. The cutoff used (85 U/dL) coincided with the 0.3 percentile and with the 36% of the mean of BTD activity of our representative sample. With this cutoff, the false positive rate of the program was 0.3%; prematurity and low birth weight accounted for the 7% of the false positive results, and the positive predictive value of the screening test was 18%. At the repeat test, the median values of BTD activity in true and false positive infants were 54.55 U/dL (range 33–78.4 U/dL) and 119.5 U/dL (range 82–325 U/dL), respectively; Figure 2. In all the newborns confirmed affected with partial BD at the initial as well as at the subsequent screening test, the enzyme activity was far lower than the 85 U/dL cutoff.

**Figure 3:**
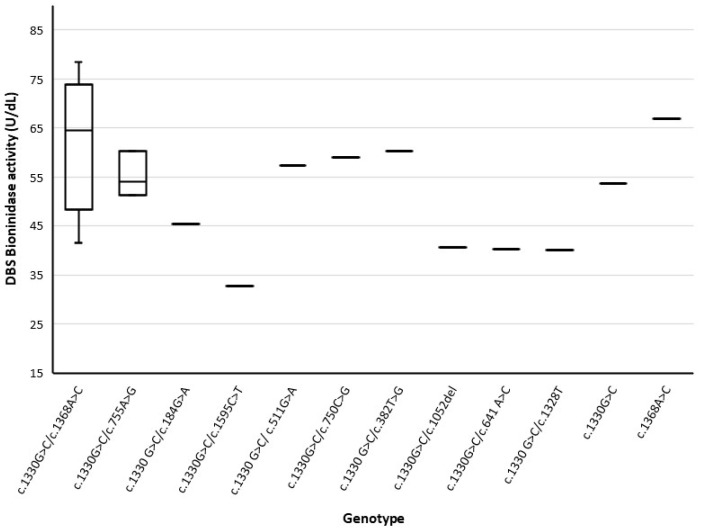
Biotinidase activity at the DBS screening test and variants in carriers and in affected infants.

According to the distribution of the BTD activity, values at the repeat test in true positives ranged between 10% and 30% of the mean of the full-term healthy newborns (mean 22.8%; range 17.5–29.8%). The lowest value was found in one infant, carrying the variant c.1595C>T (p.T532M). An overlap between the BTD activity of affected infants and of the two carriers was observed, Figure 3.

**Figure 4:**
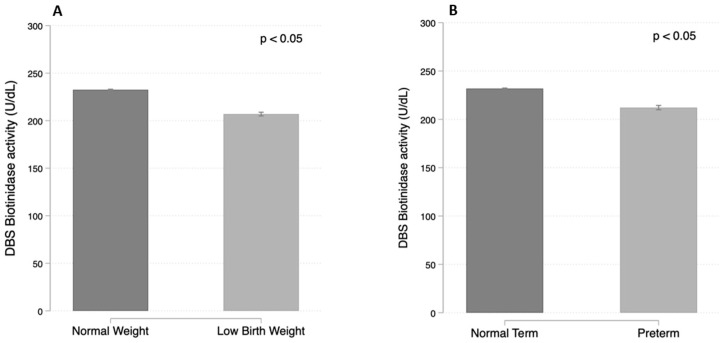
DBS biotinidase activity in relation to the birth weight (**A**) and to the pregnancy term (**B**).

In the whole newborn population, the biotinidase activity at the initial screening test positively correlated with the gestational age (ρ = 0.141; *p* < 0.001), the birth weight (ρ = 0.171; *p* < 0.001), and the age at specimen collection (ρ = 0.084; *p* < 0.001). Lower values were observed in low-birth-weight newborns (median 203.65 U/dL; interquartile range 75.75) and in preterm babies (median 213.7 U/dL; interquartile range 75.8) (Figure 4).

**Figure 5:**
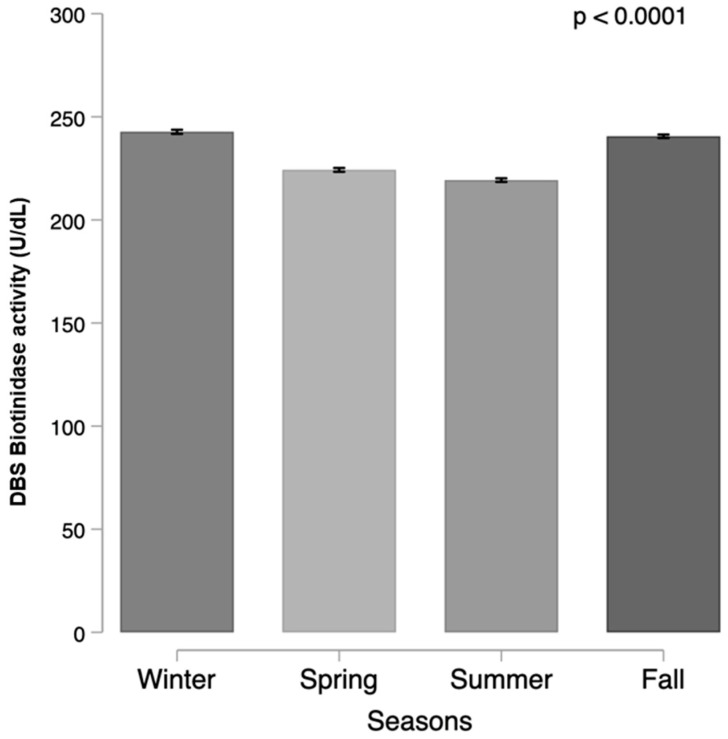
Mean DBS biotinidase activity in relation to the birth season.

Seasonal changes of BA were observed, with values significantly lower during the summer (mean 219.3 ± 53.7 U/dL,) compared to those in all the other seasons (242.7 ± 51.1, 224.3 ± 52.63442, and 240.5 ± 50.5/dL in winter, spring, and fall, respectively); Figure 5.

**Figure 6:**
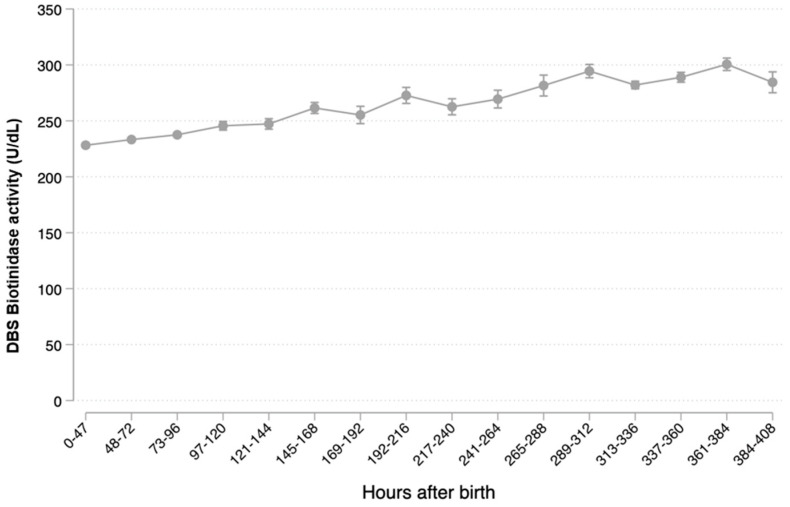
Mean biotinidase activity in DBS vs. the age at specimen collection.

A progressive increase in the mean BTD activity was observed with advancing age at collection, Figure 6.

## 4. Discussion

Newborn screening for BD allows the early diagnosis and treatment of affected individuals, substantially changing the natural history of the disease [3]. Individuals with either profound or partial BD are usually identified with biotinidase activity assay on DBS. Newborn screening is not meant to be the method to determine definitive diagnosis, and serum quantitative enzymatic testing combined with molecular analysis of the *BTD* gene should be performed to confirm the suspect. Nevertheless, the correlation between genotype and biochemical/clinical phenotype is not always consistent; ambiguous enzymatic test results, overlap of enzyme activity between carriers and individuals with partial BD, and different results in two consecutive serum samples, leading to divergent classification of the phenotype, have been reported [3,19]. Therefore, genetic analysis of the proband and of the family members is required to define the status of an infant suspected to have BD since some mutations are consistently reported as associated with profound or partial enzyme deficiency. In a recent study addressing the correlation between genotype and biochemical phenotype (DBS and serum biotinidase), accordance was observed in 93.8% of the cases. The authors also underline that newborns with near-normal enzyme activity and a variant allele were reported to present symptoms under stress conditions [20]. In this retrospective study, covering the first 3 years of experience of a regional reference laboratory in Italy, we reported the outcome of our screening program and reviewed the performance parameters of our algorithm. We have found fair accordance between the screening test results and the molecular analysis since all the variants detected in our population, except the new one, were previously reported to be associated with partial BD. In our study, positive newborns had enzyme activities on DBS ranging between 10% and 30% of the mean of our “normal” neonatal population. An overlap of DBS biotinidase activity was found between the two heterozygous and the compound heterozygous infants. No cases with low biotinidase activity and genotype suggestive of profound BD were found. Based on the biotinidase activity assay on DBS and on the molecular analysis, we could report an incidence of partial BD of 1:1649 newborns screened. Nevertheless, we are aware that, according to the guidelines, the newborn screening test should not be used to discriminate between profound and partial BD, despite the above-mentioned pitfalls in serum biotinidase assay and the potential informative role of molecular analysis in unambiguously determining “an individual’s enzyme deficient status” if a molecular variant database has been established [5]. Indeed, it is worth remembering that our algorithm did not include, initially, the serum biotinidase assay as a confirmation test and this is a limitation of the study. Therefore, if we take into account only the three of our cases with biochemical and genetic confirmation, the incidence of partial BD in our population is 1:8797. We are going to perform serum biotinidase assay as soon as newborns attend the clinical reference center for the follow-up. Pending that, we can purely speculate a higher incidence of partial BD in our population; in fact, it would be surprising that 12 of 13 newborns harboring variants consistently associated with partial BD but not biochemically confirmed had normal biotinidase activity on serum assay even assuming a genotype–phenotype concordance of 50%.

Although the sample size should be larger for such a rare disease, we report an incidence of partial BD that is in the high range of what has been reported in the literature.

The incidence of BD varies across countries due to population-specific factors, such as high rates of consanguinity [21,22], but also due to the increasing inclusion of BD in the national screening programs as well as to variations in the screening program strategies (methods of BTD activity assay, cutoff, and algorithms). Pivotal studies reported that the incidence of the disorder was about 1:112,271 newborns for profound and 1:129,282 for partial BD [8]. In subsequent studies, a higher incidence was reported in the USA (1:101,779 and 1:16,533 newborns for profound and partial BD, respectively) [23], Canada (1:15,000 combined profound and partial BD [19]), Brazil (between 1:22,861 and 1:13,909 combined profound and partial) [24], Greece (1: 4508 partial BD), and the Netherlands (1:6100 to 1:8200 neonates for partial and profound BD, respectively [25]). In Italy, the following incidences have been reported: combined profound BD between 1:61,000 and 1:6215 and partial BD between 1:137,236 and 1:7172 [12,26]. In a recent work, a higher incidence has been reported: 1:58,757 profound DB and 1:6677 partial BD [13].

In our population, genetic analysis revealed that the p.D444H variant was present in 17 infants, confirming its high prevalence among Europeans affected with partial BTD. Subjects homozygous for this variant have 50% of mean normal BTD activity, while heterozygous with variants for profound BD have 10–30% of the mean normal enzyme activity that is the range of the partial BD form [4,27]. The most common variants were compound heterozygote p.D252G/p.D444H and p.D444H/p.Q456H found, together, in 50% of our affected infants. The variant p.Q456H has been frequently described in European population. In one infant, we found the p.F250L variant not previously described and likely pathogenic by the prediction tools.

We reviewed the key performance parameter of our screening program to verify the efficacy of our algorithm. The selection of the appropriate cutoff is the main challenge of every screening program and is aimed at minimizing the false positive as well as the false negative rate. The positive predictive value of the screening test for BD varies widely according to the assay (colorimetric or fluorescent based) and to the cutoff used. In our screening program, we used a fluorescent-based assay that allows a “quantification” of BTD activity and a fixed cutoff to discriminate between negative and positive newborns. The cutoff chosen at the set-up of the program was confirmed according to the distribution of biotinidase activity in our large representative sample of “normal” population, to the seasonal variations, and to the data of true positive newborns. Our preliminary cutoff of 85 U/dL corresponds to the 0.3 percentile of the distribution of BTD activity value of our population, and that is what we still use in our protocol. In all the true positive newborns at the initial as well as at the subsequent screening test, the enzyme activity was far lower than 85 U/dL, thus suggesting that this value should avoid missing newborns at risk for partial DB with an acceptable false positive rate. Integrating the data of the screening test with those coming from the clinical follow-up of the cases could lead in the future to a revision of the cutoff and of the program algorithm.

With the 85 U/dL cutoff, the false positive rate of the program was 0.3%, which is far lower than that for congenital hypothyroidism in our experience; prematurity and low birth weight accounted for the 7% of the false positive results. Based on the molecular analysis, the positive predictive value of the screening test was 18%.

In a large study, the positive predictive value of the screening test for BD was 6.4%, with a false positive rate 2 to 10 times lower than that of tests for phenylketonuria and galactosemia [28]. In subsequent and more recent studies, positive predictive value between 30 and 86% have been reported [20,24,28]. Again, variations in the positive predictive value of the NBS tests are related to a series of pre-analytical (age at specimen collection, prematurity, specimen storage, and transportation) and analytical factors. Among the latter are the type of assay and the cutoff used. Indeed, when the main target of NBS is the profound BD, a lower cutoff is suggested to reduce the false positive [25]. Otherwise, a more conservative and age-related cutoff is advocated to avoid missing partial BD [29].

In our population, as expected, BTD activity was positively correlated with gestational age, birth weight, and age at specimen collection. In the confirmed case of partial BD, a slight increase in enzyme activity was observed in the repeat test, but the values were still far lower than the cutoff. The lower mean BTD activity observed during the summer period confirms the influence of external factors on DBS enzymatic screening tests. In fact, despite the detailed recommendations on transport and storage, it cannot be excluded that DBS are exposed to high temperatures and humidity. Attention to the external factors (sample collection, handling and storage, reagent lot, and seasonal variations) is always required when dealing with tests that measure enzyme activity [30].

All infants affected with partial BD detected by our screening program were asymptomatic and are still followed once a year in an outpatient clinic. Treatment of profound BD is lifelong administration of biotin 5–20 mg daily, reducing the dose as the children grow [6]. Treatment of partial BD was initially controversial, and still concerns remain in indications, dosage, and duration. Recently, a recovery of BTD activity has been demonstrated with increasing age and mainly in subjects carrying the p.(Asp444His) variant on at least one allele. Thus, re-evaluation of BTD activity at the age of 5 years has been suggested in order to adjust or stop biotin supplementation [31].

We initially recommended treatment with biotin 5 mg daily only during stressing conditions, while in the past year, we started treatment as soon partial BD was confirmed.

## 5. Conclusions

In the first 3 years of our regional newborn screening program, we found an incidence of partial BD that is in the high range of what has been reported in the literature. We found a good correlation between the result of the screening test and the molecular analysis, since all the newborns referred for low DBS biotinidase activity carried variants already reported as associated with partial BD. A novel variant, likely pathogenetic, was found in one infant. The screening program showed high accuracy, an acceptable false positive rate, and a positive predictive value. Correlation between some pre-analytical factors (gestational age, birth weight, age at specimen collection, and season of birth) and DBS biotinidase activity was observed.

## Figures and Tables

**Table 1:** BTD gene analysis of the infants with positive newborn screening test.

Number of Patients	Allele 1	Allele 2
5	c.1330G>C (p.D444H)	c.1368A>C (p.Q456H)
3	c.1330G>C (p.D444H)	c.755A>G (p.D252G)
1	c.1330G>C (p.D444H)	c.1595C>T (p.T532M)
1	c.1330G>C (p.D444H)	c.511G>A (p.A171T)
1	c.1330G>C (p.D444H)	c.184G>A (p.V62M)
1	c.1330G>C (p.D444H)	c.750C>G (p.F250L) *
1	c.1330G>C (p.D444H)	c.382T>G (p.F128V)
1	c.1330G>C (p.D444H)	c.1052 del (p.T351K)
1	c.1330G>C (p.D444H)	c.641 A>C (p.N214T)
1	c.1330G>C (p.D444H)	c.1328T (p.F443S)
1	c.1330G>C (p.D444H)	-
1	c.1368A>C (p.Q456H)	-

* New variant.

## Data Availability

Data supporting results are contained within the article.
